# In Memoriam: The Scientific Legacy of Dr. Katherine T. Peter

**DOI:** 10.1021/acs.est.5c15202

**Published:** 2025-12-24

**Authors:** David M. Cwiertny, Edward P. Kolodziej, Gabrielle P. Black, John Kucklick, Ruth Marfil-Vega, Andrew D. McEachran, Benjamin J. Place, Jessica L. Reiner, Alix E. Rodowa

**Affiliations:** † 4083 University of Iowa, Departments of Civil & Environmental Engineering and Chemistry, Iowa City, Iowa 52242, United States; ‡ Center for Urban Waters, Tacoma, Washington 98421, United States; § Interdisciplinary Arts and Sciences, 43339 University of Washington Tacoma, Tacoma, Washington 98421, United States; ∥ Department of Environmental Engineering, University of Washington, Seattle, Washington 98195, United States; ⊥ The Environmental Forensics Group, LLC, Duxbury, Massachusetts 02332, United States; # The National Institute of Standards and Technology, Chemical Sciences Division, Charleston, South Carolina 29412, United States; ∇ Shimadzu Corporation, Tokyo 101-8448, Japan; ○ Agilent Technologies, Inc., Santa Clara, California 95051, United States; ◆ 10833 The National Institute of Standards and Technology, Chemical Sciences Division, Gaithersburg, Maryland 20899, United States

**Keywords:** water treatment, water quality, high-resolution mass spectrometry, nontargeted analysis, early onset colorectal cancer, in-memoriam, Katherine T. Peter

## Abstract

The untimely passing of Dr. Katherine T. Peter on November 4, 2024, was profoundly felt by the many scientists, colleagues, friends, mentors, and mentees whom she touched throughout her career. To everyone who knew Kathy, two things stood out: her deep love for her husband and son, Jason and Oliver Dorn, and her unwavering passion for the meticulous development of research tools for public and environmental health. Kathy obtained a B.S. in Chemical Engineering from Washington University and a Ph.D. in Civil and Environmental Engineering from the University of Iowa before conducting postdoctoral research at the Center for Urban Waters (University of Washington—Tacoma) and the National Institute of Standards and Technology. She authored over 25 peer-reviewed publications focused on water treatment technologies, water quality characterization, and high-resolution mass spectrometry tools. Her contributions were recognized with numerous honors, including the James J. Morgan Environmental Science & Technology Early Career Award and the Best Practices for Non-Targeted Analysis Outstanding Service Award. Kathy excelled as a researcher and collaborator, a skilled coordinator of multidisciplinary science, a passionate advocate for community outreach and engagement, and a gifted communicator who made complex ideas accessible—all while maintaining the life she loved as a dedicated mother, wife, quilter, and hiker.

## Graphical abstract

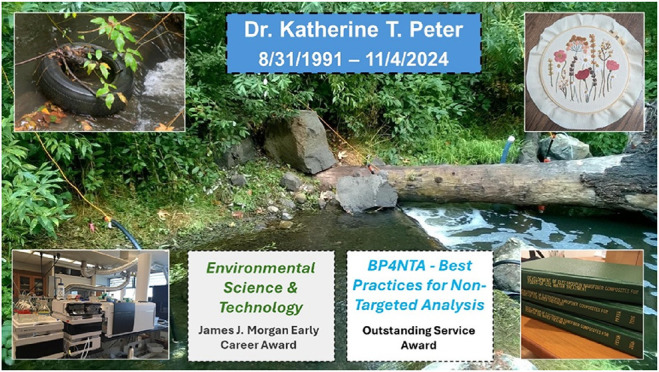

## Introduction

We sadly report the untimely passing of Dr. Katherine T. Peter (affectionately known as “Kathy”) on November 4, 2024, as a result of early onset colorectal cancer (EOCRC). During her all too short research career, Kathy worked on novel materials for water treatment and the development of advanced mass spectrometry tools for environmental characterization, treatment, and human health assessment. Here, those of us so touched and inspired by Kathy’s brilliance as a friend, collaborator, and researcher seek to recognize and communicate her scientific and personal accomplishments as we celebrate her intellectual legacy. Simply put, she was the very best of us.

## Undergraduate and Doctoral Research at Washington University and the University of Iowa: Environmental Nanotechnology as a Tool for Water Treatment

Kathy’s career started in the Department of Energy, Environmental and Chemical Engineering at Washington University in St. Louis. There, she conducted undergraduate research in the laboratory of Dr. John Fortner, broadly in the area of environmental nanotechnology. Her contributions led to her first peer-reviewed publication in *ES&T Letters*, exploring the photo-oxidation of hydrogenated fullerenes in water.^1^ Kathy was an accomplished student, earning a perfect 4.0 GPA while attaining her Bachelor of Science in Chemical Engineering (valedictorian of the College of Engineering while double majoring in Spanish) and playing for the university softball team.

Kathy then pursued a Ph.D. in Civil and Environmental Engineering at the University of Iowa through support from the NSF Graduate Research Fellowship. At Iowa, Kathy continued her work on environmental nanotechnology, crafting her thesis around the application of electrospun nanofibers in point-of-use water treatment. Her work was motivated by the numerous challenges confronting Iowa’s private well owners, where over 300,000 vulnerable consumers fall outside of Safe Drinking Water Act protections and often need multifunctional, broad-spectrum purification technologies in a small device footprint.

Kathy’s doctoral research developed simple yet elegant synthetic approaches for high-performing nanomaterials. Her first lead-author paper focused on the application of carbon nanofibers as a sorbent material.^2^ It was notable for its use of phthalic acid, which functioned as a porogen, to increase the surface area, flexibility, and durability of the resulting nanofiber layer. Kathy also discovered that integration of surface-segregating surfactants like quaternary ammonium compounds resulted in nanofiber composites that were surface-enriched with both quaternary ammonium groups (ion exchange sites) and metal oxide nanoparticles (sorption and complexation sites).^3^ This “single pot” synthesis provided a creative way to fabricate multifunctional materials without postsynthesis processing. Kathy successfully applied these materials in flow-through systems for arsenic treatment, a contaminant that frequently impairs private wells. Her final thesis publication was a clever integration of her prior work, where she demonstrated how leachable surfactants could act as porogens to add surface area and pore volume in functionalized polymer nanofibers for greater removal of lead and other dissolved metals.^4^

Kathy’s doctoral work was distinguished by its balance of fundamental discovery with translational science and engineering. Kathy creatively designed materials to exhibit not only high reactivity but also material strength that made them practically applicable under the constraints of point-of-use treatment systems. Collectively, her thesis research advanced the application of engineered nanomaterials in drinking water treatment, including widely cited publications and two awarded US patents^5,6^ while illustrating her exceptional capability as a material scientist and engineer.

## Postdoctoral Work at the Center for Urban Waters: High-Resolution Mass Spectrometry to Characterize Water Quality

Following completion of her Ph.D. and husband-to-be Jason’s deployment to Joint Base Lewis-McChord in Washington State, Kathy arrived at the Center for Urban Waters (CUW; Tacoma, WA) in December 2016 as a postdoctoral scholar and research scientist. Although Kathy had limited prior experience with water quality characterization, she fully embraced the intellectual and conceptual challenges inherent to high resolution mass spectrometry (HRMS), especially the data science, statistical and analytical tools needed to make sense of the flood of complex data that a HRMS instrument generates. Kathy found these challenges highly motivating–finally finding a tool that could match and fully engage her powerful and flexible intellect.

During Kathy’s first stint at CUW (2016–2019), almost the entire research group was focused on the compelling question of: what was killing Pacific Northwest coho salmon during fall rainstorms? To understand observations of recurrent acute mortality, the CUW lab was using environmental mass spectrometry tools to characterize and track the chemical composition of roadway runoff, urban stormwater, and associated receiving waters. This unifying theme and focus on discovery science initiated an integrated effort to apply comparative HRMS analysis to roadway runoff, small creek receiving waters, and associated treatment systems. It also motivated the development of source tracking and fingerprinting tools that could be used to compare and characterize water quality degradation and dynamics during the storm events where coho salmon would perish. This was all done in conjunction with parallel toxicology studies led by Dr. Jen McIntyre (Washington State University) and Dr. Nat Scholz (NOAA-NMFS).

Kathy quickly became an intellectual and organizational leader and driving force of many research and analytical studies to better understand urban water quality and associated chemodynamics. For example, CUW was working with citizen science and community groups to enable sampling of receiving waters where symptomatic coho salmon were observed during rain events. Kathy enhanced and initiated collaborations with several community groups, including Puget Soundkeeper, the Thornton Creek Alliance, and especially the Miller-Walker Community Salmon Investigation located on Miller Creek in Burien/Normandy Park WA, USA. Here, on Miller Creek, Kathy began what would be a fruitful and insightful long-term investigation of source and receiving water composition during storm events. In Peter et al. 2018, Kathy first correlated coho mortality events to roadway chemicals via mass spectrometry data, including a statistical association between coho mortality and tire rubber leachate chemicals.^7^ This study, reflecting a paired toxicology-environmental sampling study design, critically focused the research team’s attention on the role of tires and tire rubber as an important, and poorly understood, contributor of abundant aquatic contaminants. Additionally, this study made compelling linkages to the occurrence of tire rubber-derived chemicals with toxic attributes during coho mortality events. Beginning with this work, the team’s focus largely shifted to understanding the environmental implications of tire rubber chemicals, an end point that can be strongly traced to Kathy’s excellent and novel analytical data.

In 2017, Dr. Jen McIntyre demonstrated that tire rubber leachate was lethally toxic to juvenile coho salmon, leading to a series of studies where Tian et al. identified 6PPD-quinone as the primary causal toxicant for stormwater-linked coho salmon mortality events.^8^ This study communicated the previously unknown environmental risks of PPD-quinones as abundant toxic contaminants arising from vehicles and environmentally dispersed tire rubber residues. Over time, this study is expected to lead to the development of safer tires as toxic antioxidants such as 6PPD are replaced with (ideally) nontoxic alternatives. Kathy was a key contributor and coauthor to Tian et al., as well as several related studies focused on characterizing roadway runoff and urban receiving waters, defining the chemical composition of tire rubbers and associated leachates, understanding the formation of 6PPD-quinone on tire rubber, and evaluating linkages between stormwater and ecosystem health and contaminant dynamics.^9–12^

Due to Kathy’s involvement, the water quality infrastructure and enthusiastic citizen science collaborations have led to multiple water quality and toxicology studies at Miller Creek which is representative of small, roadway runoff-impacted watersheds. After the initial 2018 Miller Creek publication, Kathy worked to define the chemodynamics of roadway runoff impacts on small riverine watersheds, demonstrating that tire chemicals can be abundant enough to be transport-limited environmental contaminants and defining exposure periods and sampling strategies needed to better understand transient water quality degradation during storm events.^13^ Successively, these dynamics were linked to end points such as biological decline,^14^ as well as data and model development to better predict roadway contaminant concentrations and transport dynamics in urbanizing creeks,^15^ and finally, a long-term study focused on 6PPD-quinone dynamics in Miller Creek over a multiyear, multiseason sampling campaign.^16^ This set of studies and projects led to Miller Creek, and its community, becoming one of Kathy’s favorite places on earth, and a place where she made an especially meaningful scientific contribution to improved water quality and ecological health.

These studies highlighted an opportunity to use nontargeted analysis (NTA; broadly defined as the characterization of the chemical composition of any given sample without the use of *a priori* knowledge regarding the sample’s chemical content) to accomplish source tracking, fingerprinting, and source apportionment (i.e., estimating the contribution of sources of organic contaminants to surface water) end points. These efforts at CUW started by tracking and identifying roadway chemicals in both water and fish tissues, demonstrating the inherent bioavailability and abundance of several roadway contaminants.^17^ In parallel with the development of quantitative liquid chromatography tandem mass spectrometry methods,^18,19^ Kathy began working to quantify the proportion of roadway runoff in Miller Creek solely using NTA data. Initial estimates of runoff contributions are found in Peter et al. (2018), but the full development of a highly novel and powerful technique for HRMS-based source apportionment was presented in Peter et al. 2019, where Kathy developed and validated the use of “dilution curves” of known sources to apportion multiple sources in complex mixtures, even down to impacts of <1% (*v/v*).^7,20^ While not yet widely appreciated or used, this technique is quite powerful, and represents a highly original and flexible use of unidentified NTA features to better understand environmental pollution. Subsequent studies not only applied^21^ and automated but also expanded^22^ these HRMS-based fingerprinting and apportionment capabilities.

Throughout her many studies focused on mass spectrometry, Kathy remained an engineer whose focus remained on problem-solving and management of complex environmental problems. The application of her analytical chemistry capabilities to applied engineering is exemplified by Peter et al., where Kathy used HRMS and LC/MS/MS techniques to evaluate the *in situ* treatment performance and optimization of a novel engineered hyporheic zone designed to improve water quality within urbanized creeks.^23^ Several new techniques and novel assessment capabilities based upon NTA data were demonstrated, showing that relatively short and transient subsurface hyporheic flow paths can rapidly sequester hydrophobic contaminants and improve water quality. This study, and related studies focused on stormwater treatment^24–26^ highlighted her practical engineering and problem-solving skills for stormwater and roadway runoff management.

## Postdoctoral Work at the National Institute of Research and Technology (Charleston, SC): Novel Methods to Identify and Track Sources of Persistent Organic Pollutants and Modernizing Standard Reference Materials

In 2019, Kathy was awarded a National Research Council Postdoctoral Research Fellowship and was posted to the National Institute of Standards & Technology in Charleston, SC. There, she continued applying her expertise using NTA for source apportionment of organic contaminants in surface water.

Kathy recognized that NTA had the potential to increase the robustness of source apportionment by vastly increasing the numbers of observable chemical features in water samples. The benefit of this approach is an increased likelihood of observing multiple organic chemicals and identifying ones scaling proportionally with hydraulic mixing. Concurrently, NTA allows for the identification of novel contaminants or transformation products associated with specific sources and can provide information regarding the chemical attributes of sources missed by the targeted chemical analysis approach.

Kathy’s contributions to the application of NTA and targeted analysis to source apportionment in water were impactful. These approaches used real world samples mixed in the laboratory and then analyzed by NTA to both detect source chemical profiles and determine which detections scaled proportionally with mixing ratios.^20^ By doing so, Kathy was able to delineate unique source “fingerprints” and estimate source contributions within 0.82- and 1.4-fold of actual dilutions. The approach was creative, meticulous, and included a thorough error and sensitivity analysis. In Du et al., the team expanded the use of NTA, deriving the chemical fingerprints of urban water sources associated with road runoff and sewage outfalls.^21^ Sources were successfully distinguished based on both unique chemicals and unique proportions of shared chemicals. At NIST, Kathy expanded this approach to include mixtures of per- and polyfluoroalkyl substances (PFAS), a ubiquitous and notoriously complex class of compounds.^27^ Apportioning sources for PFAS using targeted approaches is hampered by a lack of authentic standards for most PFAS. Kathy serially mixed two aqueous film forming foams (AFFF) containing a diverse mixture of PFAS with water types of increasing natural matrix complexity to identify AFFF-specific subsets of PFAS whose concentrations changed proportionally to AFFF concentration and were reliably detected. Using relative concentrations and PFAS fingerprints, NTA-derived source estimates were up to 90% of the actual concentration after internal standard normalization. The method shows great promise for use in real-world situations where there are both complex PFAS sources and high backgrounds of natural organic matter. Software tools published by Hu et al. facilitated the curation of NTA data and use in source tracking and estimation.^22^

Kathy’s work challenged the assumption that wastewater is the dominant source of contaminants entering urban watersheds. In some of her later work, Kathy using targeted analyses in the relatively well-studied San Francisco Bay estuary to demonstrate the usually overlooked importance of stormwater as a contaminant source.^28^ The study is noteworthy due to the diversity (roadway runoff tracers, organophosphate esters, and PFAS) and number (154) of contaminants monitored. The routine detection of 68 compounds shows the importance of including stormwater in addition to wastewater in broad watershed assessments.

Leveraging her strong background in NTA, Kathy also began applying NTA to increase the chemical information available on NIST Reference Material certificates. Her efforts resulted in the first example of providing NTA data for reference materials, specifically for reference materials 8690–8693 Per- and Polyfluoroalkyl Substances (PFAS) in AFFF Formulation I–IV.^29^ Kathy’s contributions will ultimately lead to the timely and necessary modernization of NTA data inclusion for stakeholder use of such materials.

## Leadership in Professional Working Groups: Advancing Best Practices in the Non-Targeted Analysis Community

In addition to her many impactful research accomplishments, Kathy contributed to the scientific community via leadership in professional working groups. Kathy was an early member of Best Practices for Non-Targeted Analysis (BP4NTA), a working group formed at a U.S. Environmental Protection Agency workshop, and soon became a key member of this international, interdisciplinary organization of scientists focused on creating a community of practitioners and establishing best practices and standards for HRMS analysis.^30^

BP4NTA initially worked to establish reference content to support NTA practitioners and a glossary of common terms. Uniting language and common concepts across a diverse group of researchers and reaching consensus is a laborious and often thankless job, and yet Kathy stepped up to lead the effort to describe performance metrics for NTA. Her contributions and leadership were essential to creating the first BP4NTA Web site and the associated reference content and glossary.^31^ This resource continues to support novice and expert users of NTA techniques. As part of this effort, Kathy recognized the need for improved guidance for assessing the performance of NTA studies. She co-led an effort to define existing capabilities across three types of NTA (sample classification, chemical identification, and chemical quantitation), culminating in a critical publication for the NTA community.^32^ Importantly, this work highlighted key caveats and areas for additional research and where new tools should be developed to improve the accuracy of performance assessments (e.g., ability to define identifiable chemical space). Kathy also worked with a multidisciplinary team on the proposed ChemSpace Tool;^33^ this project, in addition to setting the foundation for ChemSter, highlights Kathy’s ability to engage with diverse stakeholders and spark collaborations that move entire fields forward.

Kathy’s ability to recognize critical gaps in the NTA field and to develop creative data tools is further evidenced by her leadership in developing the Study Reporting Tool with BP4NTA.^34^ Often NTA details are poorly described in the literature or elements of quality control are lacking, which challenges the reproducibility and reliability of NTA results and conclusions. To address this gap, Kathy co-led the development of the Study Reporting Tool as an easy-to-use interdisciplinary guide for reporting NTA methods and results. As part of this effort, she developed and tested a scoring system to facilitate assessment/communication of study reporting quality for peer review. The Study Reporting Tool is currently in use by the NTA community-at-large and has also garnered attention from other -omics groups grappling with similar issues of standards reporting.

Recent undertakings by BP4NTA include developing study planning guidance to support researchers in NTA study design to be mindful of study scope and results communication. While an ongoing effort, Kathy’s influence and interest in high standards for NTA study design and rigorous results evaluation will help guide development of these new tools for continued standardization and benchmarking of NTA. Kathy’s unique gift for uniting communities, distilling complex and often conflicting viewpoints, and forging a path for consensus-based progress is evident by her many contributions to BP4NTA. Given the breadth and significance of Kathy’s contributions to BP4NTA and the greater NTA community, she was the inaugural recipient of the BP4NTA Outstanding Service (BOS) Award in 2023, which has since been renamed the Katherine T. Peter BOS Award to inspire future NTA researchers.

## Inspiring Scientists and Working with the Public: Community Engagement and Impact

Kathy’s dedication to community engagement was evident throughout her career, evidenced by her work with students, citizen science groups, and professional organizations to bridge the gap between research and real-world impact. She was passionate about environmental education, delivering presentations to elementary, middle, and high school students. Whether explaining stormwater pollution to fourth graders in Charleston or communicating the chemistry of salmon mortality to students in Seattle, WA, and Montgomery County, MD, Kathy made complex scientific concepts both understandable and exciting. Her enthusiasm for science education also extended to university students, where she served as a guest speaker at the University of Puget Sound and helped to educate the next generation of environmental analytical chemists.

Beyond the classroom, Kathy played a pivotal role in community-driven science. She led the CUW’s engagement with citizen science groups in the Seattle/Tacoma area, fostering collaborations with local volunteers to take an active role in environmental research. She organized stormwater sampling efforts, coordinated reporting of salmon distress, and facilitated community discussions about the impact of pollution on aquatic life. This commitment to public outreach and scientific achievement was recognized by many, helping to earn her the James J. Morgan Environmental Science & Technology Early Career Award in 2021.^35^ Through her work and career, she not only advanced her field but put great effort into making science accessible, relevant, and inspiring to people of all ages, ensuring a long-lasting and impactful legacy as both a researcher and communicator.

## Lessons from Kathy’s Life for Environmental Engineers and Chemists: Needs

Kathy passed away from colorectal cancer in her early 30s, shortly after becoming a first-time mother and without any compelling risk factors for EOCRC. Her diagnosis and experience with cancer itself highlights several significant and motivating research needs, notably including the etiology of EOCRC and possible linkages to environmental chemical exposures, as well as methods to reduce medical biases in maternity and oncologic care. Over the past two decades, the incidence of EOCRC, defined as colon cancer cases in people under the age of 50, has doubled.^36^ While genetics, diet, obesity, alcohol consumption, tobacco use, and stress are all established risk factors for EOCRC, the reasons for this dramatic rise remain unknown. Emerging theories include detection bias via the increased use of colonoscopy screening, changes in the gut microbiome, and early life exposures during fetal development, childhood, adolescence, and young adulthood.^37^ The birth cohort hypothesis for EOCRC posits that individuals born in the year 1960 or later are at a greater risk of developing EOCRC due to cumulative lifetime chemical exposures compared to those of older generations,^38^ a timeline that coincides with the development and use of so many new synthetic chemicals our collective community is working to track throughout the environment.^39^ For example, of ∼1000 chemicals evaluated, the International Agency for Research on Cancer (IARC) has classified over 100 as “carcinogenic to humans”.^40^ Among these, some dietary additives (e.g., nitrates, synthetic food dyes, etc.),^37^ air pollutants,^41^ pesticides,^42^ endocrine disrupting chemicals (EDCs),^43^ microplastics^44^ have been implicated with increased EOCRC rates.

Understanding the etiology of EOCRC is itself a complex challenge requiring new tools to expedite exposure research and approaches to link complex exposures to apical outcomes. Kathy’s contributions to NTA are themselves examples of these types of research outcomes as scientists and medical practitioners are increasingly using nontargeted, multiomic, and molecular epidemiology approaches to characterize the molecular basis of EOCRC. Untargeted metabolomics mirrors NTA and is being used to better understand exposure-related disruptions of host metabolism, and chemical exposures have been correlated to metabolic responses related to disease etiology.^45,46^ Integrative omics techniques (genomics, epigenomics, transcriptomics, and proteomics) have been used to survey human colorectal tumors, providing important information for the precision treatment of EOCRC.^47^ Multiomics analyses have also reported metabolite-microbiome correlations in individuals with EOCRC, with the potential to be further developed into biomarkers for screening and early prevention.^48^ To allow mining of multiomics data relevant to EOCRC, scientists have assembled the CRCDB (A Comprehensive Database for Integrating and Analyzing Omics Data of Early onset and Late-onset Colorectal Cancer; http://crcdb-hust.com), a database containing multiomics data for 785 EOCRC, 4898 late-onset colorectal cancers, and 1110 normal control samples from tissue, whole blood, platelets, and serum exosomes.^49^ These efforts draw on advanced and emerging chemical analysis techniques and successful collaboration of scientists across sectors and expertise areas, clear examples of what Kathy herself was dedicated to as a researcher.

Equally important to understanding chemical exposures on EOCRC pathogenesis, effective medical decision-making is crucial to diagnosis, prognosis, and overall patient well-being. During Kathy’s battle with EOCRC, she became a patient advocate, particularly for pregnant women navigating the medical system. She was awarded an honorable mention for her submission “None of My Mom Friends Are Dying” in the *Pulse* writing contest “On Being Different”.^50^ She recognized the biases that can impact medical decision-making and intentionally published her essay in an online platform that reaches both patients and medical professionals. EOCRC is difficult to diagnose and is therefore more likely to be diagnosed at an advanced disease state compared to traditional colorectal cancer.^51^ Testing/diagnostic delays thus result from low suspicion of cancer at younger patient ages.^52^ In addition, women often face gender disparities within the healthcare system. For example, women’s reports of pain are often taken less seriously by health care professionals than men’s.^53^ Young women are particularly disadvantaged with regard to delays in EOCRC diagnosis.^54^ Diagnosis of EOCRC in pregnant women is especially complicated given the many natural physiologic changes associated with pregnancy.^55^ Anchoring (i.e., initial information tends to have greater influence than later information) and confirmation (i.e., the tendency to seek or interpret information in ways that support current beliefs and dismiss information that is inconsistent with those beliefs)^56^ biases are two common cognitive biases known to affect medical decision-making and likely play a role in the often delayed diagnosis of EOCRC. The Society for Maternal-Fetal Medicine recently published some strategies to debias clinical decision-making, including self-education, medical education, departmental activities, and workplace modification.^57^ Kathy’s willingness to bravely share her experience with EOCRC further communicates awareness of EOCRC to both clinicians and patients, sheds light on barriers to obtaining a timely diagnosis, and is a call to action for researchers and medical professionals alike.

Throughout her lifetime, Kathy made meaningful contributions as a researcher and to her local communities. She leaves an impressive scientific legacy that challenges and motivates all of to us to continue the work that she began—improving water treatment for the general public, striving to uncover chemical causes of environmental harm, identifying and tracking sources of chemicals, promoting excellence in scientific pursuit, giving back to communities by making science accessible, impactful, and exciting, and moving through our world with grace and purpose. A shining example for us all; an example of what we do, and why it all matters.

## ¶

John Kucklick is now retired.

## Abbreviations

(6PPD-quinone): 2-anilino-5-[(4-methylpentan-2-yl)­amino]­cyclohexa-2,5-diene-1,4-dione
(AFFF): aqueous film-forming foam
(BP4NTA): Best Practices for Non-Targeted Analysis
(BOS Award): BP4NTA Outstanding Service Award
(CUW): Center for Urban Waters
(EOCRC): early onset colorectal cancer
(EDC): endocrine disrupting chemical
(HRMS): high resolution mass spectrometry
(IARC): International Agency for Research on Cancer
(LC/MS/MS): liquid chromatography tandem mass spectrometry
(NIST): National Institute of Standards and Technology
(NOAA-NMFS): National Oceanic and Atmospheric Administration - National Marine Fisheries Service
(NTA): nontargeted analysis
(PFAS): per- and polyfluoroalkyl substances
(PPD): phenyl phenylenediamine
(RM): reference material
(SRT): Study Reporting Tool
